# Possible Tracheal Relaxant and Antimicrobial Effects of the Essential Oil of Ethiopian Thyme Species (*Thymus serrulatus* Hochst. ex Benth.): A Multiple Mechanistic Approach

**DOI:** 10.3389/fphar.2021.615228

**Published:** 2021-04-05

**Authors:** Najeeb Ur Rehman, Mohd Nazam Ansari, Tesfay Haile, Aman Karim, Khalil Y Abujheisha, Syed Rizwan Ahamad, Faisal Imam

**Affiliations:** ^1^ Department of Pharmacology and Toxicology, College of Pharmacy, Prince Sattam Bin Abdulaziz University, Al-Kharj, Saudi Arabia; ^2^ Department of Pharmacognosy, School of Pharmacy, College of Health Sciences, Mekelle University, Mekelle, Ethiopia; ^3^ Department of Biological Sciences, National University of Medical Sciences, Rawalpindi, Pakistan; ^4^ Faculty of Natural and Health Science, Al Zaytoonh University of Science and Technology, Salfeet, Palestine; ^5^ Central Laboratory, Department of Pharmaceutical Chemistry, College of Pharmacy, King Saud University, Riyadh, Saudi Arabia; ^6^ Department of Pharmacology and Toxicology, College of Pharmacy, King Saud University, Riyadh, Saudi Arabia

**Keywords:** antimicrobial, asthma, bronchodilatation, Ca^++^ channel blocker, phosphodiesterase inhibitor, *Thymus serrulatus*

## Abstract

The genus Thymus is traditionally used for the treatment of hyperactive airways complaints. The purpose of the current study is to investigate the potential tracheal relaxant effect and possible mechanism(s) of the essential oil of *Thymus serrulatus* (TS Oil) in isolated guinea pig tracheal tissues. The essential oil was obtained from the fresh erial parts of *Thymus serrulatus*, and its phyto-components were identified by GC-MS analysis. Guinea pig tracheal preparations were used for testing the tracheal relaxant effect of TS Oil with the determination of the mechanism(s) involved in this relaxation. GC-MS findings reveal that terpenes, fragrance constituents, saponins, and higher fatty acids are present in TS Oil. In isolated guinea pig trachea, TS Oil inhibited carbachol (CCh, 1 µM) and K^+^ (80 mM)-induced contractions in a pattern similar to that of dicyclomine. TS Oil, at 0.3 mg/ml, shifted parallel CCh-curves towards the right, followed by a non-parallel shift at higher concentration (1 mg/ml), thus suppressing maximum response in the same manner as produced by dicyclomine. Pretreatment of tissues with TS Oil (1 and 3 mg/ml) also produced a rightward shift of Ca^++^ concentration-response curves (CRCs) in the same manner as caused by verapamil. Further, TS Oil at low concentrations (0.3 and 1 mg/ml) shifted isoprenaline-induced inhibitory CRCs towards the left and increased cAMP levels in isolated tracheal homogenates similar to papaverine, a phosphodiesterase (PDE) inhibitor. In the antimicrobial assay performed by the agar well diffusion method, TS Oil was found most active against *Candida albicans* and *Staphylococcus aureus* where the zone of inhibition measured was 28 mm. Additionally, there was little difference between standard strains of gram-positive and gram-negative bacteria. However, methicillin-resistant *S. aureus* (MRSA) showed a small zone of inhibition as compared to standard strains (22 mm). From these results, it can be concluded that the essential oil of *T. serrulatus* has the potential to produce antimicrobial effects while causing tracheal relaxation mediated possibly by anticholinergic effects, Ca^++^ channel blockade, and PDE inhibition whereas additional mechanism(s) cannot be ruled out.

## Introduction

Asthma is a condition of chronic obstruction of airways, inflammation, and mucus production, which results in shortness of breath and/or cough. It usually occurs in the evening and/or in the early morning as tracheal sensitivity to many stimulators increases at these times. Various immune cells contribute to the exacerbation of asthma including mast cells, eosinophils, and T-cells (Guo et al., 2018). Reports show that some selected species of bacteria and fungi such as *Klebsiella pneumoniae*, *Pseudomonas eruginosa*, *Staphylococcus aureus*, and *Candida albicans* and methicillin-resistant *S. aureus* (MRSA) are also involved in causing airways diseases such as laryngitis, pharyngitis, and tracheitis (Costa et al., 2015; Gileles-Hillel et al., 2015). However, certain infections are difficult to treat. Further, the therapeutic options available to address respiratory disorders have several limitations including cost and side effects. Thus, alternative remedies, such as aromatic plants, have been used to treat these disorders and possibly have bronchodilatory and antimicrobial effects (Costa et al., 2015; Cheesman et al., 2017).

*Thymus serrulatus* Hochst. ex Benth (Lamiaceae), a much-branched perennial subshrub that grows in the Afromontane and Afroalpine zones of Ethiopia and Eritrea (Damtie and Mekonnen, 2015). In Ethiopia, it is found in the northern highlands of Semien Shoa, Tigray, and Wollo areas (Melka et al., 2016, Damtie et al., 2018). It is known by the local communities as Tosign in Amhara and Tesni/Thasne in Tigray region of Ethiopia (Damtie et al., 2017).

*T. serrulatus* is an endemic medicinal plant traditionally used for various disease conditions including flue (Meresa et al., 2017) and cough (Melka et al., 2016). In various scientific studies the plant has been found active against various bacterial and fungal strains (Damtie and Mokonen, 2016), possess diuretic and antihyperlipidemic (Melka et al., 2016; Getachew, 2018). Also, the smooth muscle relaxant effect (vasodilatory) of the plant was observed in an *ex vivo* study by (Geleta et al., 2015).

In different part of the world, various species of thymus (*T. linearis*, *T. vulgaris*, and *T. serphyllum*, etc.) are known for their traditional us in the treatment of respiratory disorders such as bronchitis, asthma, pertussis, laryngitis, tonsillitis, and cough caused by common colds (Begrow et al., 2010; Alamgeer et al., 2018). The essential oil of various thyme species were reported to be active against airways disorders (Horváth and Ács, 2015). The essential oil obtained from *T. serrulatus* collected from different Ethiopian localities reported the presence of active ingredients such as thymol, carvacrol, p-cymene, γ-terpenene, and rosmarinic acid (Asfaw et al., 2000; Damtie et al., 2018). Further, terpenes, such as thymol and carvacrol, that have been found as major constituents in essential oil-containing plants have also been reported to possess bronchodilatory effects and reduce inflammation in airways disorders (Zhou et al., 2014). Further, the findings reported by (Ahmad et al., 2010) show that thymol and carvacrol have antibacterial and antifungal activities in addition to multiple other biological effects. Based on the above mentioned traditional and scientific evidences of various thymus species and their essential oils active against various airways disorders, we in this study aimed to chemically characterize, evaluate the possible tracheal relaxant effects of *T. serrulatus* in *ex vivo* model along with antimicrobial property of *T. serrulatus* essential oil.

## Materials and Methods

### Plant Material and Extraction

A sample of fresh erial parts of *T. serrulatus*, was collected during the flowering stage (October 2018) from the Amba Alaje mountain area located in Amba Alaje District (12° 59′ 54.4″N, 39° 32′ 52.3″E) South Tigray, Ethiopia. After it was collected, the plant material was identified according to the Flora of Tropical Africa (Daniel, 1900) and authenticated by botanist, Dr Getinet Masresha from the Department of Biology, University of Gondar and for further reference, the specimen has been deposited at the herbarium of the University with voucher number TH-001/2011.

After collection, the fresh aerial parts of *T. serrulatus* were cut into small pieces and subjected to hydro-distillation (water distillation) for 3 h by using a Clevenger-type apparatus. Hydro-distillation was performed 11 times until enough amount of sample was collected. The obtained essential oil was dried using anhydrous sodium sulfate and stored in tightly closed vials at 4°C until further testing and analysis (Asfaw et al., 2000). The calculated essential oil yield was expressed in percentage (% v/w), based on the weight of the fresh plant material. A stock solution of the obtained oil was prepared by mixing 300 uL of oil (≈300 mg) with 700 uL of distilled water containing 10% DMSO to obtain a stock concentration of 300 mg/ml. The formula, “density = mass/volume (d = m/v),” was used while calculating the mass of *T. serrulatus* oil where the density of oil was found equal to one. Further dilutions were prepared from this stock solution (300 mg/ml) where the final bath concentration of DMSO obtained was <1% in bronchodilatory and antimicrobial experiments.

### Chemicals and Animals

Chemicals used in this study include atropine sulfate, carbachol (CCh), dicyclomine, isoprenaline, verapamil, and papaverine were procured from Sigma (St. Louis, MO, United States). The cAMP enzyme immunoassay kit used to estimate cAMP was procured from Sigma-Aldrich Co., United States. It is worth noting that all other chemicals used were of analytical grade.

Guinea-pigs (either sex, weighing 510–560 g) were housed at the animal house located at College of Pharmacy, PSAU, Saudi Arabia in a controlled environment maintained at 25 ± 2°C. The animals were provided with free access to water and commercial standard diet. All experiments were done as per the guidelines of the Institute of Laboratory Animal Resources, Commission on Life Sciences, National Research Council (National Research Council, 1996). The study was approved by Bio-Ethical Research Committee (BERC), Prince Sattam Bin Abdulaziz University (Approval number BERC-004–12–19).

### GC-MS Analysis

The extracted oil of *T. serrulatus* was analyzed by the Perkin Elmer gas chromatograph (Clarus 600) coupled with a Perkin Elmer (Clarus 600 T) mass selective detector. Subsequently, 1 µL of the aliquots of the extracts were injected into Elite 5-MS capillary column (30m × 250 µm I. D, 0.25 µm film thickness; Perkin Elmer, Shelton, CT, United States) in the splitless mode of 1:20. The column temperature was kept at 40°C during sample injection and was raised to 150°C for 2 min having rate of 10°C/min and further increased to 300°C held for 2 min. The injector temperature was at 280°C, inlet line temperature at 220°C, and source temperature was 220°C. The total run time was 32 min. Helium was used as the carrier gas at a flow rate of 1.0 ml per min. Detection was carried out by using MS detection in electron-ionization mode using a single quadrupole detector and full-scan monitoring mode (m/z 40–600).

Each individual phyto-constituent was recognized on the basis of its retention time and mass spectrum included in the National Institute of Standards and Technology (NIST, 2005) and Wiley 2006. Out of 90 identified compounds, most of the compounds were identified from the matrix, and some came from the column itself. The compounds that occupied a negligible percentage of the total areas were discarded. Finally, about 50 compounds with match factor 60% were added in the method.

### *Ex-vivo* Studies on Isolated Guinea-Pig Trachea

The trachea was isolated from guinea pig sacrificed by cervical dislocation and exsanguinated. Approximately, 2–3 cartilaginous rings, constituting one tracheal tissue, were opened by a longitudinal cut opposite to the smooth muscle layer. The opened tissue was then mounted in a 20 ml organ tube containing Kreb’s solution (37°C) and bubbled with carbogen. A tension equal to 1 g was constantly maintained in each of the tracheal tissues during the experiment. During the equilibration of tissue for 1 h, the Krebs solution in the bath was replaced every 15 min, while CCh (1 µM) and K^+^ (80 mM) were repeatedly used to stabilize the tissue until constant contractions of each agonist were achieved. After stabilization, the relaxant effect of TS Oil was determined by adding it cumulatively every 10 min to get the concentration-dependent effects. Tracheal contraction and relaxation were recorded by using isometric transducers attached to emkaBath with IOX software (France).

To investigate the possible effect of the plant oil to exhibit inhibitory action on Ca^++^ channels, the tracheal tissues were depolarized with high K^+^ (80 mM). K^+^ > 30 mM is known to depolarize the tissue by opening of voltage-gated Ca^++^ channels and/or other transporters and channels such as; Ca-Na exchanger and TRP channels and thus, causing smooth muscle contractions (Wray, 2010). Meanwhile, the substance inhibiting high K^+^-induced contraction might in some way leads to a reduction in the Ca^++^ influx through these pathways and might be considered as a Ca^++^ channel blockers (Hussain et al., 2008). In the next step, to endorse the Ca^++^ channel blockade (CCB) effect of TS Oil, the tracheal tissues stabilized in normal Kreb’s solution were immersed in Ca^++^-free Kreb’s solution containing EDTA (0.1 mM) where the tissues were incubated for 30 min to remove Ca^++^ from the tissue. Thereafter, the Ca^++^-free Kreb’s solution was replaced with K^+^-rich and Ca^++^-free Kreb’s solution. After incubation of tracheal tissue for an hour in K^+^-rich and Ca^++^-free Kreb’s solution, Ca^++^ induced contractions were recorded to obtained control CRCs. When the Ca^++^-CRCs were found to be superimposable, the tracheal tissues were incubated with TS Oil for 1 h. The CRCs of Ca^++^ were repeated in the presence of increasing concentrations (1 and 3 mg/ml) of the TS Oil and the results were compared using verapamil (0.03 and 0.1 µM) as standard (positive control).

To test whether the tracheal relaxant effect of the TS Oil also involves any additional mechanism(s), such as anticholinergic like effects, the CRCs of CCh were constructed by adding CCh in cumulative manner to the bath and the tissues were washed with fresh Krebs solution after achieving CCh maximum peaks. The CCh CRCs were repeated in the presence of pre-incubated tissues with increasing concentrations (0.3 and 1 mg/ml) of TS Oil for 1 h (Shah and Gilani, 2010).

Usually, plant materials that produce tracheal relaxation also show inhibitory actions on the PDE-enzyme, hence the method described by (Gilani et al., 2005) was followed to assess the presence of possible PDE inhibitory effects in our plant essential oil. Briefly, inhibitory CRCs of isoprenaline were constructed against CCh-induced contractions in the absence (control) and in the presence of TS Oil.

### The cAMP Estimation

Phosphodiesterase’s decreases the levels of active cAMP and cGMP by metabolizing them to inactive forms AMP and GMP. Therefore, one of the mechanisms for an agent that causes increase in the levels of tissue cAMP and GMP is considered PDE inhibition. Hence, to test TS Oil for the possible PDE inhibitory action, cAMP contents of the trachea were measured by enzyme immunoassay using the direct cAMP enzyme immunoassay kit (Sigma-Aldrich, Saint Lewis, United States). For this purpose, two sets of tracheal tissues, one set was contracted with CCh alone (control) and second set was contracted with CCh followed by inhibition with increasing concentrations of TS Oil (treated). Both types of the tracheal sets were immediately removed from the organ bath and were frozen in liquid nitrogen. After grinding the tissues into fine powder, homogenization was done immediately in 0.1 M HCl (10 volumes) followed by centrifugation (600 g, 10 min) at 22°C. The supernatant was extracted from centrifuged samples and diluted with 0.1 M HCl and stored at −80°C till the time of determination of cAMP. The tissue concentration of cAMP content was expressed as pmol/mg protein.

## *In-vitro* Antimicrobial Activity

### Microbial Strains

TS Oil was tested for the possible antimicrobial effect against both, the reference strains from the American Type Culture Collection (ATCC) and National Collection of Type Culture (NCTC) and clinical strains. These strains have role in respiratory diseases and include *Klebsiella pneumoniae*, *Pseudomonas aeruginosa*, *Staphylococcus aureus*, and Candida albicans while the clinical pathogens include *K. pneumoniae* and methicillin-resistant *S. aureus* (MRSA). All the tested pathogen strains were routinely grown aerobically at 37°C.

### Antimicrobial Assay

The screening activity of TS Oil was performed by the agar well diffusion method. A suspension of tested microbes equivalent to 0.5 McFarland was cultivated in Mueller-Hinton agar (MHA, Scharlau). TS Oil (50 µL) and 1% DMSO as control were added in each well, and the plates were incubated at 37°C for 24 h and zones of inhibitions were measured.

#### Determination of Minimum Inhibitory Concentration (MIC)

MIC assays of TS Oil were performed by the broth dilution method by following standard guidelines as detailed in (Clinical and Laboratory Standards Institute (CLSI), 2016). Nine dilutions of TS Oil, ranging from 12.5to 150 μg/ml, were prepared. Selected strain of each pathogen (10 µL) was inoculated into each individual concentration of TS Oil. For testing sterility, Mueller-Hinton broth (MHB, Scharlau) was tested alone as control whereas vehicle (DMSO) effect was tested on untreated bacteria inoculated on MHB alone and with 1% DMSO as growth control. After 24 h incubation at 37 °C, the MIC of the TS Oil was calculated based on the lowest concentration of antimicrobial agent that inhibited visible growth. Minimal microbicidal concentration (MMC) in 24 h incubation was evaluated by the sub-culture of 10 µL from the broth dilution in Mueller-Hinton agar, Scharlau. All assays were run in duplicate to authenticate and statistically analyze the data.

### Statistical Analysis

Values are represented as mean ± standard error of the mean (SEM) and the median effective concentrations (EC_50_) with 95% confidence intervals (CI). The statistical parameters applied were Student’s *t*-test or Repeated Measures ANOVA followed by Bonferroni’s post-test for comparisons of CRCs with their respective controls. Differences were considered statistically significant at *P* < 0.05. CRCs were analyzed by non-linear regression using the GraphPad program (Graph Pad, San Diego, CA, United States).

## Results

### Essential Oil Yield (%)

Hydro-distillation of the erial part of the *T. serrulatus* gave 0.09% (v/w, on a fresh weight basis) pale yellow essential oil with a characteristic odor.

### GC-MS Analysis

**FIGURE 1:**
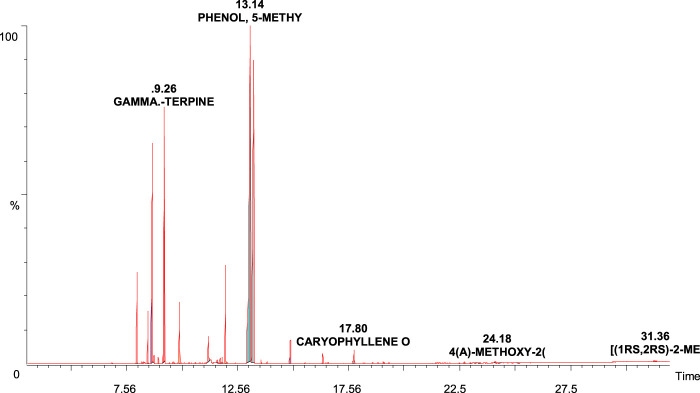
GC-MS chromatogram of crude extract of *Thymus serrulatus* essential oil (TS Oil).

Mainly flavoring components like terpenes, sesquiterpenes, and higher fatty acids were found to present in TS Oil; however, it tested negative for other classes (Figure 1). The details of all compounds and their retention time (RT) are shown in Table 1.

**TABLE 1:** List of Phytocomponents present in *Thymus serrulatus* essential oil (TS Oil).

S. No	Phytocomponents	RT	RI	m/z	Mol. Formula	Area%	Area	Nature of compound
1	Beta-myrcene	8.03	994	136	C_10_H_16_	2.84	12873136	Monoterpene hydrocarbon
2	3-Octanol	8.24	1008	130	C_8_H_18_O	0.08	348035	Aliphatic alcohol
3	L-phellandrene	8.33	1011	136	C_10_H_16_	0.10	435135	Cyclic monoterpene hydrocarbon
4	Alpha terpene	8.52	1023	136	C_10_H_16_	1.2	5448806	Isomeric monoterpene
5	Cumene	8.72	1028	120	C_9_H_12_	17.1	77818448	Aromatic hydrocarbon
6	DL-limonene	8.75	1032	136	C_10_H_16_	0.03	157735	Cyclic monoterpene
7	Cis ocimene	8.79	1036	136	C_10_H_16_	0.16	741465	Aliphatic monoterpene
8	3,7 dimethyl 1,2,3 octatriene	8.98	1039	136	C_10_H_16_	0.17	761899	Alpha menthrene
9	Gamma terpenene	9.26	1067	136	C_10_H_16_	15.7	71396784	Isomeric monoterpene
10	Cis beta terpineol	9.49	1108	154	C_10_H_18_O	0.02	113118	Monoterpene alcohol
12	Linalool oxide	9.71	1125	170	C_10_H_18_O_2_	0.02	102576	Monoterpenoid
13	Linalool	9.95	1132	154	C_10_H_18_O	1.34	6082895	Terpene alcohol
14	Caryophyllene diepoxide	10.36	1158	236	C_15_H_24_O_2_	0.02	76865	Sesquiterpene
15	Trans pinene hydrate	10.66	1169	154	C_10_H_18_O	0.01	37629	Flavouring compound
16	4-terpineol	11.24	1193	154	C_10_H_18_O	0.77	3497233	Monoterpene alcohol
17	3-cyclohexene-1-methanol	11.47	1208	112	C_7_H_12_O	0.02	93833	Alcohol
18	Adamantene 1-carboxylic acid	11.53	1215	304	C_15_H_20_O_3_N_4_	0.07	316037	Cyclic aliphatic acid
19	(+)- Alpha terpineol	11.65	1221	154	C_10_H_18_O	0.14	628060	Monoterpene alcohol
21	Thymyl methyl ether	11.86	1236	164	C_11_H_16_O	0.23	1042000	Aromatic benzene
22	2-isopropyl-5-methyl-1-methoxybenzene	12.02	1248	164	C_11_H_1_6O	4.53	20546648	Aromatic benzene
24	Thymol	13.10	1325	150	C_10_H_14_O	34.31	155602208	Oxygenated monoterpene
25	Carvacrol	13.30	1336	150	C_10_H_14_O	19.57	88752792	Oxygenated monoterpene
26	Thymyl acetate	13.62	1363	192	C_12_H_16_O_2_	0.11	489107	Monoterpenoid
27	Carvacryl acetate	13.89	1392	192	C_12_H_16_O_2_	0.07	304335	Monoterpene
28	Trans-caryophyllene	14.92	1465	204	C_15_H_24_	0.510	2322053	Bicyclic sesquiterpene
29	Alpha-humulene	15.59	1475	204	C_15_H_24_	0.010	61979	Monocyclic sesquiterpene
30	Gamma-cadinene	15.88	1538	204	C_15_H_24_	0.010	19317	Sesquiterpene
31	Caryophyllene oxide	17.80	1710	220	C_15_H_24_O	0.280	1270515	Oxygenated sesquiterpene
32	Humulene oxide	18.21	1746	220	C_15_H_24_O	0.010	23422	Monocyclic sesquiterpene
34	Hybridalactone	18.64	1784	316	C_20_H_28_O_3_	0.02	84609	Eicosanoid
35	Caryophylla-2 (12),5-dien-7-one	18.89	1805	218	C_15_H_22_O	0.01	31488	Bicyclic sesquiterpene
36	(+)- beta costol	19.11	1820	220	C_15_H_24_O	0.02	79546	Naphtha derivative
37	Lidene oxide	19.37	1838	220	C_15_H_24_O	0.01	29605	Oxirene derivative
38	9-Octadecanoic acid	29.72	2858	282	C_18_H_34_O_2_	0.05	229602	Fatty acid

(RT), Retention Time; (RI), Retention Index.

### *Ex-vivo* Effects on Guinea-Pig Trachea

#### Effect on CCh and High K^+^-Mediated Contractions

**FIGURE 2:**
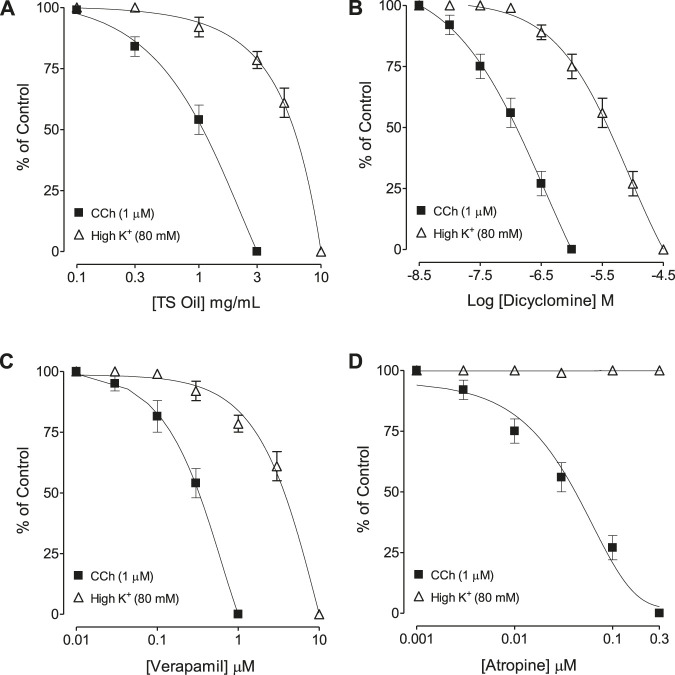
Concentration-response curves showing comparison of **(A)** *Thymus serrulatus* essential oil (TS Oil), **(B)** dicylomine, **(C)** verapamil, and **(D)** atropine for the inhibitory effect against carbachol (CCh) and high K + -induced contractions in isolated guinea-pig tracheal preparations. Values shown are mean ± SEM of 3–5 individual experiments conducted on the isolated tracheal tissue preparations from 3 to 5 different guinea-pigs.

In the in-vitro experiments shown in Figure 2A, TS Oil inhibited CCh (1 µM) and K^+^ (80 mM)-induced contractions. A higher potency was observed against CCh with EC50 measurement of 0.86 mg/ml (0.64–1.24, *n* = 4) as compared to high K^+^ where the obtained EC50 value was 5.24 mg/ml (4.22–6.34, *n* = 5). A positive control drug, dicyclomine, behaved in a pattern similar to TS Oil against CCh and K^+^ (80 mM) with respective EC50 values of 0.48 µM (0.38–0.52, *n* = 4) and 5.16 µM (4.86–6.62, *n* = 4) (Figure 2B). On the other hand, unlike the plant oil and dicyclomine, verapamil was found more potent against K^+^-induced contractions with an EC50 value of 0.44 µM (0.32–0.58, *n* = 3) as compared to CCh-mediated contractions with EC50 value of 5.27 µM (4.55–6.42, *n* = 3) as seen in Figure 2C. Results of atropine, as shown in Figure 2D, show only selective relaxation against CCh with EC50 measurement of 0.06 µM (0.04–0.07, *n* = 3), while zero efficacy was observed against high K^+^.

#### Effect on CCh-Curves

**FIGURE 3:**
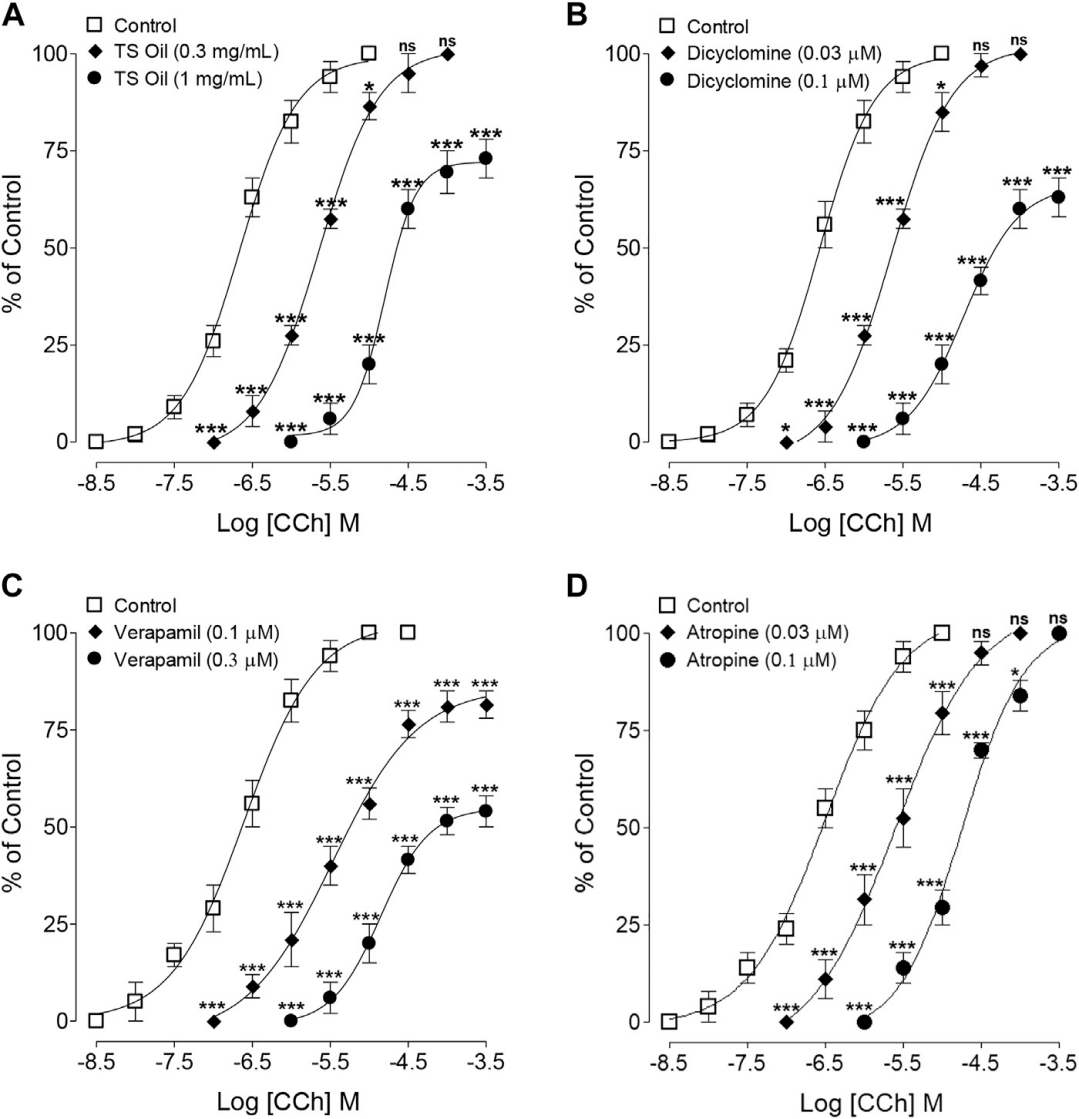
Concentration-response curves of carbachol (CCh) in the absence (control) and presence of increasing concentrations of **(A)** *Thymus serrulatus* essential oil (TS Oil), **(B)** dicyclomine, **(C)** verapamil, and **(D)** atropine in isolated guinea-pig tracheal preparations. Values shown are mean ± SEM of 3–4 individual experiments conducted on the isolated tracheal tissue preparations from 3 to 4 different guinea-pigs. *p < 0.05, **p < 0.01, and ***p < 0.001 shows comparison of the mean of CCh-mediated contractions in the pretreated tissues with TS Oil **(A)**, dicyclomine **(B)**, verapamil **(C)** and atropine **(D)** with the respective mean of CCh-mediated contractions in control (untreated) tracheal tissues (Repeated Measures ANOVA, followed by Bonferroni post-test).

In the anticholinergic, subtype antimuscarinic assay, in the absence of TS Oil, CRCs of CCh achieved maximum contraction (100%) with EC50 values of 0.21 µM (0.16–0.27; *n* = 5) while TS Oil pre-incubation at 0.3 mg/ml produced a parallel shift in CCh-peaks toward right without affecting the maximal response (100%) with EC50 values of 2.27 µM (1.78–2.90; *n* = 5), whereas the pre-incubation of trachea with next higher concentration of TS Oil (1.0 mg/ml) reduced the efficacy of CCh peaks to 73% with obtained EC50 values of 15.18 µM (11.40–21.92; *n* = 5) as shown in Figure 3A. Similar to TS Oil, dicyclomine (0.03 and 0.1 µM) pre-incubation, exhibited a similar pattern of shift in CCh peaks (Figure 3B), while the pre-incubation of verapamil at both concentrations (0.1 and 0.3 µM) produced a non-parallel rightward shift with the suppression CCh peaks (Figure 3C). Meanwhile, atropine (0.03 and 0.1 µM) pre-incubation, caused a parallel shift CCh CRCs towards right without the suppression of CCh peaks at both tested concentrations (Figure 3D).

#### Effect on Ca^++^-Curves

**FIGURE 4:**
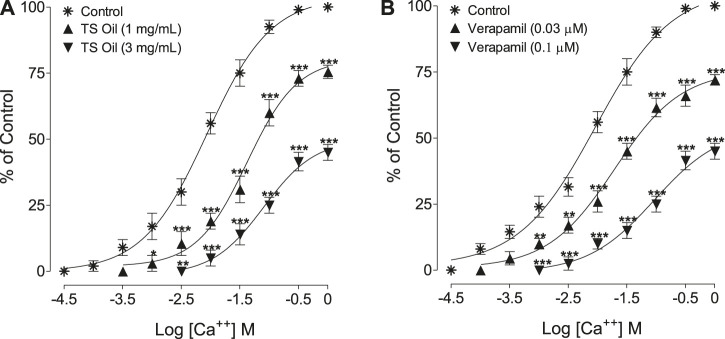
Concentration-response curves of Ca^++^ in the absence (control) and presence of the increasing concentrations of **(A)** *Thymus serrulatus* essential oil (TS Oil) and **(B)** verapamil in isolated guinea-pig tracheal preparations. The values shown are mean ± SEM of 3–4 individual experiments conducted on the isolated tracheal tissue preparations from 3 to 4 different guinea-pigs. *p < 0.05, **p < 0.01, and ***p < 0.001 shows comparison of the mean of Ca^++^-mediated contractions in the pretreated tissues with TS Oil. **(A)** and verapamil, **(B)** with the respective mean of Ca^++^-mediated contractions in control (untreated) tracheal tissues (Repeated Measures ANOVA, followed by Bonferroni post-test).

Following the preliminary results shown in Figure 2A showing effect of TS Oil against K^+^ (80 mM), the CCB like activity of TS Oil was further confirmed when the oil at both concentrations (1.0–3.0 mg/ml) produced a shift in the Ca^++^ CRCs toward right in a similar pattern of verapamil (Figures 4A,B). In the absence of TS Oil, CRCs of Ca^++^ achieved maximum contraction (100%) with EC50 values of 8.65 mM (6.47–11.56; *n* = 6) while TS Oil pre-incubation at 1 and 3 mg/ml produced a non-parallel rightward shift in with suppression of the maximal response to Ca^++^ (75%; 1 mg/ml) and (45%; 3 mg/ml) with EC50 values of 41.85 mM (26.20–66.84; *n* = 6) and 80.77 mM (37.39–174.50; *n* = 6), respectively, (Figures 4A,B).

#### Effect on Isoprenaline-Curves

**FIGURE 5:**
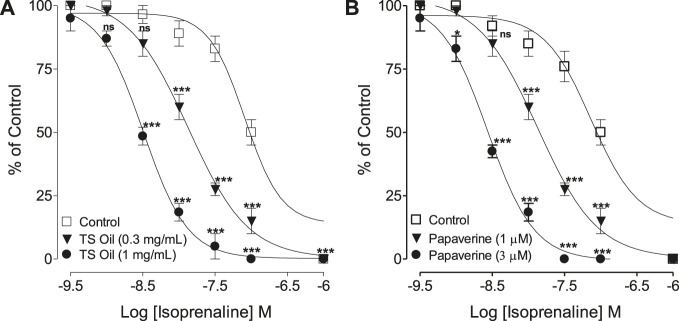
Inhibitory concentration-response curves of isoprenaline against carbachol (CCh)-induced contractions in the absence (control) and presence of different concentrations of **(A)** *Thymus serrulatus* essential oil (TS Oil) and **(B)** papaverine in isolated guinea-pig tracheal preparations. Values shown are mean ± SEM 3–4 individual experiments conducted on the isolated tracheal tissue preparations from 3 to 4 different guinea-pigs. ^ns^ *p* > 0.05, *p < 0.05, and ***p < 0.001 shows comparison of the mean of CCh-mediated inhibition by isoprenaline in the pretreated tissues with TS Oil **(A)** and verapamil, **(B)** with the respective mean of CCh-mediated inhibition by isoprenaline in control (untreated) tracheal tissues (Repeated Measures ANOVA, followed by Bonferroni post-test).

TS Oil pretreated tracheal tissues was concentration-dependent (0.3–1.0 mg/ml) and displaced the isoprenaline-induced inhibitory CRCs toward left similar to papaverine (1.0–3.0 μM) that was used as a control drug (Figures 5A,B). In the absence of TS Oil, 100% inhibition of CCh-mediated contractions was achieved by isoprenaline with EC50 values of 0.08 µM (0.05–0.12; *n* = 6) while TS Oil pre-incubation at 0.3 and1 did not affect the efficacy (100%) of the inhibitory response of isoprenaline against CCh, however significant reduction in the EC50 values [0.01 µM (0.010–0.017; *n* = 5) and 0.003 µM (0.002–0.004; *n* = 5), respectively, ] were observed (Figures 5A,B).

### Effect on cAMP Levels

**FIGURE 6:**
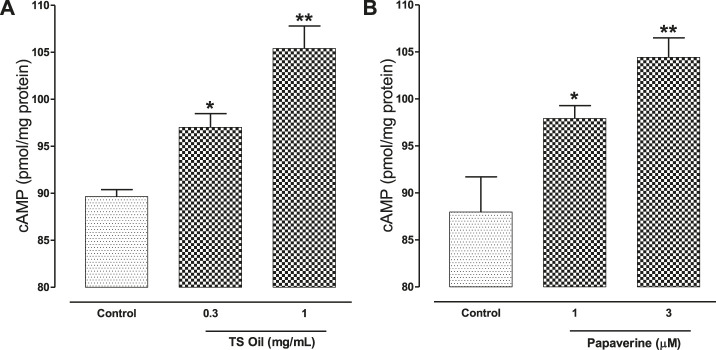
Effect of *Thymus serrulatus* essential oil (TS Oil) in carbachol (CCh)-induced contractions in the absence (control) and presence of different concentrations of **(A)** *Thymus serrulatus* essential oil (TS Oil) and **(B)** papaverine on the cyclic nucleotide content of guinea-pig trachea. Values shown are mean ± SEM of 3–4 individual experiments conducted on the isolated tracheal tissue preparations from 3 to 4 different guinea-pigs. *P < 0.05 and **P < 0.01, considered as statistically significant versus their respective controls (unpaired *t*-test).

In the cAMP estimation assay of TS Oil, the cAMP levels measured in the untreated tracheal tissues (vehicle control) were 89.65 ± 0.745 pmol of cAMP/mg protein of tissue homogenate while in pre-incubated tissues with 0.3 and 1 mg/ml of TS Oil, the cAMP levels measured were 97.02 ± 1.459 (*p* < 0.05) and 105.42 ± 2.390 (*p* < 0.01) pmol/mg protein, respectively, (Figure 6A). The positive control (papaverine) pre-incubation with 1 and 3 µM increased the cAMP levels to 97.9 3 ± 1.365 pmol/mg protein (*p* < 0.05) and 104.43 ± 2.078 (*p* < 0.01) pmol/mg protein, respectively, (Figure 6B).

### *In-vitro* Antimicrobial Effects

#### Antibiotic Susceptibility Pattern of Clinical Pathogens

The antibiotic susceptibility pattern was depicted in Table 2 where *K. pneumoniae* was found resistant to Ampicillin and Amoxicillin/clavulanate only while, MRSA showed resistance to Imipenem, Cefoxitin, Cefotaxime, Ampicillin, Penciling, Oxacillin, and Amoxicillin/clavulanate.

**TABLE 2:** Antibiotic Susceptibility patterns of Clinical Pathogens.

Bacteria	GN	IMI	FOX	CTX	AMP	PG	OX	AUG	TS	VAN	NI	CIP	CXM	CAZ	CPM	ATM
MRSA	S	R	R	R	R	R	R	R	S	S	S	S	-	-	-	-
*K. pneumoniae*	S	S	S	S	R	-	-	R	-	-	S	S	S	S	S	S

(S), Susceptible; (R), Resistant; (-), No Result; MRSA, Methicillin-Resistant *S. aureus*; *K. pneumoniae* Klebsiella pneumoniae. GN, Gentamicin; IMI, Imipenem; FOX, cefoxitin; CTX, cefotaxime; AMP, Ampicillin; PG, Penciling; OX, Oxacillin; AUG, Amox/Calv; TS, Trimethoprim/Sulfa; VAN, Vancomycin; NI, Nitrofurantoin; CIP, Ciprofloxacin; CXM, cefuroxime; CAZ, ceftazidime; CPM, cefepime; ATM, Aztreonam.

#### Screening Activity and MIC Determination

The TS Oil was found most active against *C. albicans* and *S. aureus* where the zone of inhibition was measured at 28 mm as compared to MRSA, which showed a zone of inhibition of 22 mm. Additionally, there was little difference (2–3 mm) between standard strains gram-positive and gram-negative bacteria. The vehicle (<1% DMSO) was found to be inert as no zone of inhibition was observed in the wells of the cultivated plates (data not shown).

The MIC and MMC of TS Oil against the tested microbes was depicted in Table 3. *C. albicans* showed greater sensitivity to TS Oil (MIC 75 μg/ml) as compared to gram-positive and gram-negative bacteria. Meanwhile, MRSA (MIC 125 μg/ml) was found more resistant as compared to standard *S. aureus* (MIC 90 μg/ml) and other tested microbes. Moreover, no difference (p > 0.05) was observed in the activity of TS Oil against clinical and standard strains of *K. pneumoniae* (MIC 95 μg/ml).

**TABLE 3:** Minimum Inhibitory Concentration (MIC) and Minimal Microbial Concentration (MMC) of *Thymus serrulatus* essential oil (TS Oil) vs. tested microbes and MIC of some antibiotics as per Clinical Laboratory Standard Institute (CLSI).

Tested microbes	MIC	MMC	MIC (µg/ml)							
			Gentamicin		Cefuroxime		Cefepime		Ciprofloxacin	
			S	R	S	R	S	R	S	R
*K. pneumoniae* (NCTC 9633)	95 μg/ml	105 μg/ml	≤4	≥16	≤8	≥32	≤2	≥16	≤1	≥4
*P. aeruginosa* (ATCC 10145)	105 μg/ml	115 μg/ml	≤4	≥16	−	−	≤8	≥32	≤1	≥4

*S. aureus* (NCTC 6571)	90 μg/ml	100 μg/ml	Vancomycin		Gentamicin		Azithromycin		Tetracycline	
			S	R	S	R	S	R	S	R
			≤2	≥16	≤4	≥16	≤2	≥8	≤4	≥16
*MRSA	125 μg/ml	137.5 μg/ml	−							
*K. pneumoniae	95 μg/ml	125 μg/ml																
*C. albicans* (ATCC 14053)	75 μg/ml	100 μg/ml																

*Clinical pathogens.

MIC, Minimum Inhibitory Concentration*;* MMC, Minimal microbicidal concentration; *K. pneumoniae, Klebsiella pneumoniae; P. aeruginosa, Pseudomonas aeruginosa; S. aureus, Staphylococcus aureus; C. albicans, Candida albicans;* MRSA*,* Methicillin-Resistant *Staphylococcus aureus*.

## Discussion

This study attempted to investigate the possible therapeutic uses of *T. serrulatus* essential oil in hyperactive airways disorders and its effectiveness against related microbial infections (Meresa et al., 2017). For this purpose, *T. serrulatus* essential oil extracted by hydro-distillation was tested on isolated guinea-pig tracheal tissues mounted in organ bath with physiological buffer and carbogen. The antimicrobial assay was conducted on selected microbial strains, and GC-MS analysis was done to characterize the active ingredients present in the essential oil. In the preliminary *ex-vivo* experiments, TS Oil was found active and inhibit the contractions induced by selected tracheal spasmogens such as carbachol (1 µM) and high K^+^ (80 mM) (Imam et al., 2020; Rehman et al., 2020). Experiments were further extended to know the mechanism(s) involved in the observed tracheal relaxation. In this context, it has been reported earlier that medicinal plants usually produced bronchodilatory effects by anticholinergic, Ca^++^ channel inhibition and/or phosphodiesterase inhibition (Ansari et al., 2020). To elucidate the possible involvement of these mechanism(s), the pattern of the inhibitory CRCs of TS Oil in guinea-pig isolated tracheal tissues against CCh and high K^+^ (80 mM) were critically analysed. It was clearly evident in the results that TS Oil selectively show more potency to inhibit carbachol as compared to high K^+^-induced contractions, hence we hypothesized that the tracheal relaxant effects of TS Oil are targeted at muscarinic receptors and Ca^++^ channels. Dicyclomine, an antagonist of muscarinic receptors as well as an inhibitor of Ca^++^ ion influx (Downie et al., 1977), exhibited a similar pattern of inhibition with lower EC50 values against CCh when compared with high K^+^. On the other hand, verapamil, a pure Ca^++^ channel inhibitor (Fleckenstein, 1977), was found more potent against the K^+^-induced contractions compared to CCh whereas, atropine, a muscarinic receptor antagonist (Delmendo et al., 1989), showed efficacy against CCh-induced contractions only, as expected. The observed dual effect of TS Oil, as an anticholinergic and CCB like, was reconfirmed by standard protocols suggesting the construction of the CCh and Ca^++^ CRCs, respectively, in the absence and presence of pre-incubated concentrations of TS Oil in isolated trachea. In the antimuscarinic assay, the pre-incubation of the tracheal tissue with lower concentration of TS Oil resulted in a parallel shift of CCh-curves towards right without the suppression of its maximum effect, which is a distinctive feature of a competitive antagonist, such as atropine (Arunlakhshana and Schild, 1959), whereas the next higher concentration of TS Oil produced a non-parallel displacement in CCh-curves to the right with the suppression of the maximum effect. This suppression of the maximum response suggests the presence of non-specific inhibition, similar to that observed previously and in the current study with verapamil, a Ca^++^ channel blocker (Gilani et al., 2008). When tested, dicyclomine also caused shifting of CCh-curves toward the right in a pattern similar to that of TS Oil. However, the treatment of tracheal tissues with verapamil at both, lower and higher concentrations caused a non-parallel shift of CCh-curves toward right with suppression of the maximum response. To prove our second hypothesis of possible involvement of TS Oil with Ca^++^ channels, the pre-treated tracheal tissues with TS Oil produced a rightward shift of the Ca^++^ curves with suppression of maximum response in a pattern similar to verapamil, which may indicate the Ca^++^ inhibitory like effects of TS Oil. Although TS Oil inhibited high K^+^-mediated contractions and also shifted and inhibited the Ca^++^ CRCs which indicates CCB like activity, however the production of second messengers, such as cAMP and cGMP, which, in turn, activate PKA and PKG respectively, leading to the blocking of these channels which need to be further ascertained by using a direct voltage-dependent Ca^++^ channel activator, such as dihydropyridine Bay K8644 (Allen et al., 1985).

According to (Gilani et al., 2005), medicinal plants that exhibit tracheal relaxant effects usually show PDE inhibition and this effect usually co-exists with Ca^++^ channel blocking activity. Phosphodiesterase enzymes belong to a superfamily that has a role in catalyzing the breakdown of intracellular second messenger molecules, including cAMP and cGMP; therefore, the inhibition of PDE will indirectly increase the intracellular levels of cAMP (Beshay et al., 2001). Maintaining a high level of cAMP in airways produces a bronchodilatory effect by causing the smooth muscles of tracheal tissues to relax. To explore the possible involvement of PDE-inhibition as one of the additional mechanism(s) in the tracheal relaxation, TS Oil was tested in indirect (Isoprenaline CRCs) and direct assay (cAMP estimation). In the indirect assay, isoprenaline inhibitory CRCs were plotted against CCh-induced contractions in the absence and presence of TS Oil increasing concentrations. Interestingly, the pre-incubation of TS Oil causes a concentration-dependent potentiating effect on the isoprenaline-mediated inhibitory CRCs which is considered one of the possible indications of the tissue PDE inhibition as PDE inhibitors are known to potentiate the effect of isoprenaline (Lorenz and Wells, 1983). A similar pattern of a leftward shift in the isoprenaline-mediated inhibitory CRCs was observed with papaverine, a known PDE inhibitor (Lipworth, 2005). Furthermore, in the cAMP quantitative assay, TS Oil pre-incubated tissues showed an increase in the levels of cAMP in dose-dependent manner, similar to papaverine. This further strengthens the possible involvement of PDE inhibition as one of the added mechanism(s) in the tracheal relaxation of TS Oil. Notably, cAMP concentration is also increased by the beta-2 receptor stimulation present in the airways that finally causes activation of adenylate cyclase (AC) and thus causing smooth muscles relaxation (Martin et al., 1994). The possible stimulant effect of TS Oil on beta-2 receptors might be excluded as the tracheal relaxant response was not effected in the pre-incubated tissues with propranolol, a beta receptor blocker (data not shown), however TS Oil effect on AC activation further need to be investigated. No change in the inhibitory effect was observed in the several reported studies show that anticholinergics and PDE inhibitors play an important role in the management of asthma (Barnes, 2006), but their use is limited because they cause cardiac stimulation, a serious adverse event (Nicholas, 2006; Nawarth, 1981). It’s worth mentioning that PDE enzyme type-4 (PDE-4) is selectively involved in causing the smooth muscles relaxation of the airways, therefore we recommend to establish the inhibitory role of TS Oil on PDE-4. Ca^++^ channel blockers have also been reported for their usefulness in bronchoconstriction (Twiss et al., 2002) with cardio-suppressant effect (Billman, 1992). The presence of CCB-constituent(s) in the TS Oil with antimuscarinic and PDE inhibitory like activities is possibly meant by nature to compensate the tachycardia which is generally observed when anticholinergics or PDE inhibitors if administered alone. This type of combination of mechanism(s) supports the concept of the use of natural remedies due to their synergistic and/or side-effect neutralizing components (Gilani et al., 2005). Further, such combinations in a single plant reduce the cost of therapy and offer advantages in evidence-based studies (Ernst, 2005).

Studies show the involvement of multiple microbes, such as bacteria and fungi, causing airways diseases (Gileles-Hillel et al., 2015; Cheesman et al., 2017). The infections caused by antibiotic-resistant pathogens such as MRSA are complicated and challenging to treat. It has been reported that aromatic plant essential oils exhibit an antimicrobial effect when used alone (Costa et al., 2015) or in combination with antibiotics to combat multidrug-resistant bacteria (Cheesman et al., 2017). Therefore, TS Oil was also tested for possible antimicrobial potential to sort out added role of the TS Oil in addressing infectious-related airways disorders. Interestingly, TS Oil showed antimicrobial effects against MRSA, *S. aureus*, and *C. albicans*, which might further demonstrate its therapeutic effects on microbial infections of respiratory system. Further studies are recommended to know the mechanism of the observed antimicrobial effect of TS Oil. GS-analysis of the TS Oil revealed the presence of various monoterpene hydrocarbons. These include the chemotypes of Thymus species; thymol (34.3%) and carvacrol (19.5%) that are identified as major phyto-constituents. Minor phyto-constituents like monoterpenes such as pinene, cymene, and caryophyllene oxide have also been identified. Thymol and carvacrol have been reported to possess bronchodilatory, antihistaminic, and sympathomimetic properties (Boskabady and Jandaghi, 2003). Besides antibacterial and antifungal activities (Ahmad et al., 2010), thymol and carvacrol also reduce the levels of IgE, IL-4, IL-5, and IL-13, as well as the number of inflammatory cells in the airways (Zhou et al., 2014). Hence, the observed combination of activities (tracheal relaxation and antimicrobial) in the essential oil of *T. serrulatus* might be co-related to its major phytochemical constituents—thymol and carvacrol. However, the involvement of more phytochemicals and unexplored mechanism(s) cannot be ruled out.

The conventional bronchodilators used in asthma include β2 agonist, anticholinergics and PDE inhibitors are well known for their cardiac stimulation which is a serious side effect (Undem, 2006). However, it is possible that the presence of combination of Ca^++^ channel blockers with anticholinergics and PDE inhibitory like agents, as determined in the essential oil of *T. serrulatus,* are likely to offset the cardiac stimulation usually associated with these bronchodilators, particularly when given orally.

In conclusion, the above findings show that the hydrodistilled essential oil of *Thymus serrulatus* possess a unique combination of tracheal relaxant mechanisms that include anticholinergic, Ca^++^ antagonist and PDE inhibitory-like with added antimicrobial effects against strains involved in respiratory infections. However, further detailed studies are recommended to investigate and precisely identify the molecules that cause the observed activities.

## Data Availability

The original contributions presented in the study are included in the article/Supplementary Material, further inquiries can be directed to the corresponding authors.
